# Human Salmonellosis Outbreak Linked to *Salmonella* Typhimurium Epidemic in Wild Songbirds, United States, 2020–2021

**DOI:** 10.3201/eid2911.230332

**Published:** 2023-11

**Authors:** Kane Patel, G. Sean Stapleton, Rosalie T. Trevejo, Waimon T. Tellier, Jeffrey Higa, Jennifer K. Adams, Sonia M. Hernandez, Susan Sanchez, Nicole M. Nemeth, Emilio E. Debess, Krysta H. Rogers, Aslı Mete, Katherine D. Watson, Leslie Foss, Mabel S.F. Low, Lauren Gollarza, Megin Nichols

**Affiliations:** Oak Ridge Institute for Science and Education, Oak Ridge, Tennessee, USA (K. Patel, G.S. Stapleton);; Centers for Disease Control and Prevention, Atlanta, Georgia, USA (K. Patel, G.S. Stapleton, J.K. Adams, M.S.F Low, L. Gollarza, M. Nichols);; Oregon Health Authority, Portland, Oregon, USA (R.T. Trevejo, E.E. Debess);; Washington State Department of Health, Shoreline, Washington, USA (W.T. Tellier);; California Department of Public Health, Los Angeles and Richmond, California, USA (J. Higa, L. Foss);; Association of Public Health Laboratories, Silver Spring, Maryland, USA (J.K. Adams);; University of Georgia, Athens, Georgia, USA (S.M. Hernandez, S. Sanchez, N.M. Nemeth);; Wildlife Health Laboratory, California Department of Fish and Wildlife, Rancho Cordova, California, USA (K.H. Rogers);; California Animal Health and Food Safety Laboratory, University of California Davis, Davis, California (A. Mete, K.D. Watson)

**Keywords:** Salmonellosis, *Salmonella*, Typhimurium, Salmonella enterica, bacteria, songbirds, outbreak, zoonoses, United States

## Abstract

*Salmonella* infection causes epidemic death in wild songbirds, with potential to spread to humans. In February 2021, public health officials in Oregon and Washington, USA, isolated a strain of *Salmonella enterica* serovar Typhimurium from humans and a wild songbird. Investigation by public health partners ultimately identified 30 illnesses in 12 states linked to an epidemic of *Salmonella* Typhimurium in songbirds. We report a multistate outbreak of human salmonellosis associated with songbirds, resulting from direct handling of sick and dead birds or indirect contact with contaminated birdfeeders. Companion animals might have contributed to the spread of *Salmonella* between songbirds and patients; the outbreak strain was detected in 1 ill dog, and a cat became ill after contact with a wild bird. This outbreak highlights a One Health issue where actions like regular cleaning of birdfeeders might reduce the health risk to wildlife, companion animals, and humans.

More than 1 million human illnesses result from *Salmonella* each year (*1*); ≈11% are attributed to animal contact (*2*,*3*). Humans infected with *Salmonella* can have abdominal pain, diarrhea, and fever that are often self-limiting, but more severe illness and death are possible (*4*). Animals can carry *Salmonella* in their gastrointestinal tract and the bacteria might shed intermittently in feces. 

Among wild songbirds, particularly those within the family Fringillidae (finches), salmonellosis can cause periodic widespread deaths, typically during winter, which can result in the deaths of hundreds to thousands of birds (*5*–*7*). Clinical signs in songbirds infected with *Salmonella* include reluctance to fly, anorexia, abnormal mentation, diarrhea, inability to swallow food, and sudden death (*8*,*9*). *Salmonella enterica* serotype Typhimurium is the most common serotype isolated from moribund songbirds during salmonellosis deaths (*6*,*8*,*10*–*13*). The prevalence of *Salmonella* among North American wild birds is difficult to determine because of species differences in shedding and current lack of standardization in surveillance efforts. A recent meta-analysis estimated an overall prevalence of 6.4% (n = 40,295 birds) based on 102 studies that investigated *Salmonella* in one third of wild bird species that breed in North America (*14*). However, prevalence can be highly variable, ranging from <1% to 22% depending on the species, habitat, and season examined (*5*,*14*,*15*).

Various host and environmental factors might contribute to the risk for wild bird salmonellosis outbreaks. Gatherings in large numbers at specific locations such as birdfeeders lead to increased density of birds and fecal contamination of the feeders and surrounding area, increasing transmission probability (*7*,*11*,*16*,*17*). The type of feeder also matters; platform feeders can result in fecal contamination of food, and the potential for such transmission might be increasing, given the expanding use of birdfeeders (*18*,*19*) in urban and suburban environments (*20*).

There is a strong seasonal component to avian salmonellosis. Most cases appear in the winter, when birds become more reliant on feeders (*20*) and are more physiologically stressed by colder temperatures. In addition, climate change has affected seasonal migration patterns, which might affect birds’ ability to find high-quality food and could introduce or exacerbate environmental stressors that might alter birds’ immune system function and increase their susceptibility to disease (*21*,*22*). Those factors collectively contribute to concern for the conservation of wild birds (*16*) and the possibility that infection could spill over to humans, although the magnitude of this risk is not yet well understood (*14*).

Enteric pathogens can be transmitted from wild birds to humans through multiple routes (*7*,*14*). Handling ill or dead birds or touching surfaces contaminated with bird feces, such as birdfeeders, has been linked to outbreaks of *Salmonella* infections in humans in the United Kingdom (*11*), Norway (*23*), and New Zealand (*8*). Indirect transmission through contact with companion animals that bridge the connection between humans and wild birds has also been linked to a human *Salmonella* outbreak in Sweden (*24*). Cats and dogs can serve as a source of human illness in several ways: bringing bird carcasses or feed into homes; tracking *Salmonella* into the household after contact with birds or contaminated environments; or ingesting birds, bird feces, or bird seed and shedding *Salmonella* in their own feces (*24*,*25*). Clinical salmonellosis (also known as songbird fever) in cats and dogs is characterized by fever, abdominal pain, diarrhea, vomiting, or systemic illness resulting from septicemia (*25*).

In February 2021, public health officials in Oregon and Washington, USA, reported to the Centers for Disease Control and Prevention (CDC) that 8 persons were infected with *Salmonella* Typhimurium; illness onset dates were December 2020–February 2021. Whole-genome sequencing (WGS) determined isolates collected from those patients were genetically related to one another as well as to an isolate from a pine siskin (*Spinus pinus*), a type of finch, collected in Oregon in December 2020 (*26*). To identify the source of human illness and conduct further case findings, public health partners initiated a multistate outbreak investigation. We report the findings of this investigation and provide recommendations for preventing salmonellosis as it relates to interaction with wild birds.

## Methods

### Identification of Human Cases

Cases were defined as patients infected with laboratory-confirmed, genetically related *Salmonella* Typhimurium based on core genome multilocus sequence typing (cgMLST) of WGS data (within 0–12 allele differences) with an illness onset of December 26, 2020–May 19, 2021. WGS was performed by using the Nextera XT library preparation kit (Illumina, https://www.illumina.com), followed by sequencing on the Illumina MiSeq. Sequences were shared with CDC for cgMLST analysis (*27*); all generated sequence data have been deposited in the National Center for Biotechnology Information (https://www.ncbi.nlm.nih.gov; Bioproject PRJNA230403). To identify historically related and newly identified cases during the investigation, we queried PulseNet (https://www.cdc.gov/pulsenet/index.html), the national molecular subtyping network for enteric disease surveillance at the CDC (*28*).

### Exposure Data

In the United States, state and local health officials routinely interview laboratory-confirmed *Salmonella*-infected patients with a standard questionnaire designed to collect demographic information and general food and animal exposures the week before illness onset. When patients are identified as having isolates matching an ongoing multistate outbreak, those data are shared with CDC. Because isolates from index cases were genetically related to an isolate from a wild bird, health officials collected additional information on patients’ exposures to dogs, cats, songbirds, birdfeeders, and bird feed, as well as characterized behaviors of patients’ pets, such as wildlife predation that could result in exposure to songbirds or birdfeeders. This information was shared with CDC and analyzed with SAS 9.4 (SAS Institute Inc; https://www.sas.com). We conducted descriptive analysis for each interview question, including frequency calculation for each answer on the basis of total number of responses. Using a binomial proportion test, we compared the percentage of patients with songbird, dog, or cat exposure within 7 days of illness onset to the FoodNet Population Survey, which measured the proportion of healthy persons reporting similar exposures in the 7 days before interview (*29*). This activity was reviewed by CDC and was conducted in accordance with applicable federal law and CDC policy (see, e.g., 45 C.F.R. part 46, 21 C.F.R. part 56; 42 U.S.C. §241(d); 5 U.S.C. §552a; 44 U.S.C. §3501 et seq).

### Animal Testing

Dead songbirds were collected in Oregon, Washington, and California for mortality investigation. In Oregon and Washington, dead songbirds were identified by members of the National Audubon Society with the assistance of the state public health veterinarian and were submitted to the Oregon State Veterinary Diagnostic Laboratory, where aerobic culture was performed. Additional songbirds were identified in Washington by veterinarians and submitted to the Washington State University Animal Disease Diagnostic Laboratory for aerobic culture. In California, incidents of sick and dead wild birds can be reported to California Department of Fish and Wildlife’s Wildlife Health Laboratory by the public, wildlife rehabilitation centers, and other agencies by telephone, email, or a web-based mortality reporting application (https://wildlife.ca.gov). In addition, the public can report dead birds to the California Department of Public Health for the West Nile virus surveillance program (https://westnile.ca.gov). Total reports of songbird deaths in California were compiled from those sources. 

The California Department of Fish and Wildlife submitted a total of 15 dead songbirds to the California Animal Health and Food Safety Laboratory System for postmortem examination. In brief, tissue samples of brain, muscle, thyroid/parathyroid glands, peripheral nerves, trachea, lung, heart, esophagus, crop, proventriculus, gizzard, pancreas, intestines, liver, spleen, adrenals, kidneys, and gonads were collected and immersed in 10% neutral buffered formalin, paraffin-embedded, sectioned at 4 µm, and stained with hematoxylin and eosin for histologic examination by light microscopy. Swabs of pathologic lesions affecting the oral cavity or gastrointestinal tract were collected for *Salmonella* testing by PCR and *Salmonella* culture. In addition, specimens were collected from companion animals when available during this investigation. State and local public health laboratories sequenced animal isolates by using standardized sequencing methods described previously (*27*).

Concurrently, the Southeastern Cooperative Wildlife Disease Study (SCWDS) at the University of Georgia (Athens, GA, USA) diagnosed salmonellosis in songbirds in the southeastern United States. When *Salmonella* was isolated from samples by means of aerobic culture at the Athens Veterinary Diagnostic Laboratory, isolation was confirmed and isolates serotyped at the National Veterinary Services Laboratory (*30*). Isolates from those birds were not available for genetic sequencing.

We conducted a comparison of genetic relatedness of isolates from humans and animals (when available). We consulted with wildlife experts regarding wild bird deaths and the biologic plausibility of *Salmonella* transmission routes between wildlife and humans. CDC, state partners, and wildlife experts jointly developed public communications to share recommendations to reduce illnesses.

## Results

### Human Cases

**Figure 1:**
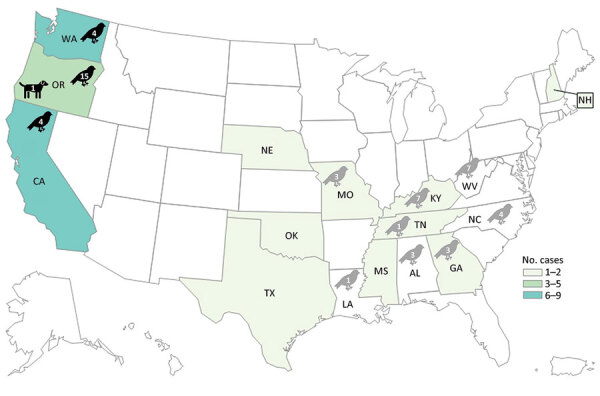
Geographic locations of human *Salmonella* Typhimurium cases in the United States, 2020–2021. Colored shading indicates number of cases by state; black bird icons indicate states that detected the outbreak strain of *Salmonella* Typhimurium from wild birds (within 0–12 allele differences based on core genome multilocus sequence typing). One isolate was obtained from a dog’s mouth wound at a veterinary hospital in Oregon (dog icon) and matched the outbreak strain. Numbers of genetically related isolates obtained from wild birds are indicated within animal icons. *Salmonella* Typhimurium was also detected in wild birds as part of the Southeastern Cooperative Wildlife Disease Study at the University of Georgia (gray bird icons); those isolates were serotyped at the National Veterinary Services Laboratory (*30*), but whole-genome sequencing was not performed to confirm relatedness to the outbreak strain.

**Figure 2:**
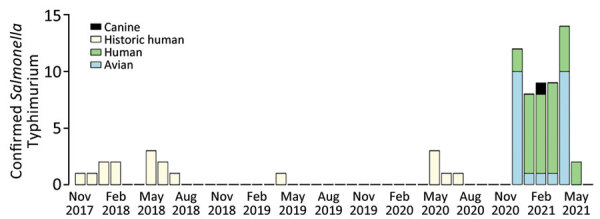
Epidemiologic curve of onset dates of human *Salmonella* Typhimurium illnesses and isolation dates of animal isolates, United States, November 7, 2017–May 19, 2021. Isolates shown are within 0–12 allele differences based on core genome mutlilocus sequence typing.

In total, we identified 30 human *Salmonella* Typhimurium cases across 12 US states (Figure 1). Twenty patients (67%) resided in the western United States (Oregon, Washington, and California). Known and estimated illness onset dates ranged from December 26, 2020, to May 19, 2021 (Figure 2). Patient ages ranged from <1 to 89 years (median 12 years); 10 patients (33%) were ≤1 year of age. Of 28 patients with information available, 14 (50%) were hospitalized, and no deaths were reported.

**Figure 3:**
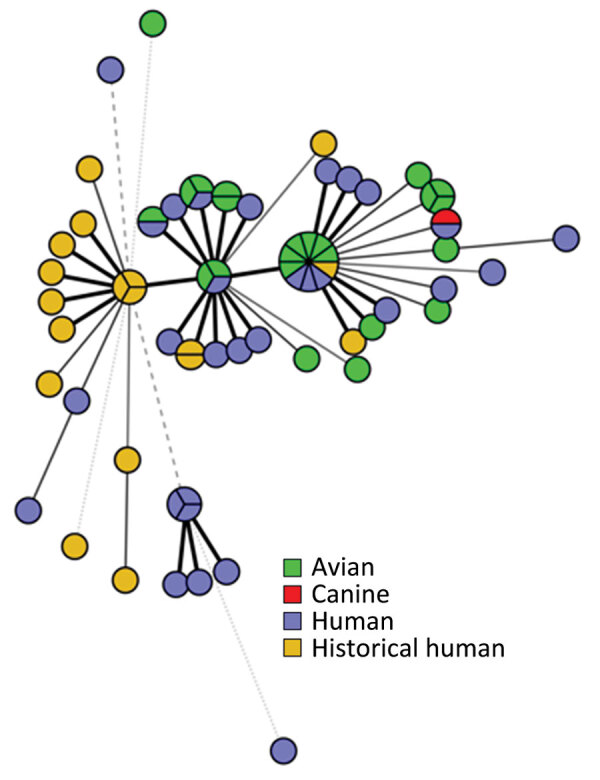
Minimum spanning tree demonstrating the genetic relatedness of human and animal isolates based on core genome multilocus sequence typing from samples obtained in the United States, 2021–2022. All sequences have an allele difference range of 0–12 alleles. Line length is proportionally scaled; the longer the line, the greater the allele difference is between the sequences. Isolates with no allelic variation are represented as slices of the same circle.

All sequences from clinical isolates were related by cgMLST within 0–12 allele differences (Figure 3). Investigators identified 18 historical clinical isolates that were closely related to the outbreak strain (within 0–10 allele differences), which were collected from Washington (n = 10), Oregon (n = 5), and Minnesota (n = 3) during November 2017–July 2020 (Figure 2). None of those isolates were linked to a specific animal or other type of outbreak, and no exposure information was available for the patients.

### Exposure Data

We interviewed 22 patients with additional detailed questions about exposure to dogs, cats, songbirds, birdfeeders, and bird feed. Of those, 14 (64%) reported having a birdfeeder on their property, 8 recalled purchasing bird seed for their feeder within 2 months before illness onset, 7 fed bird seed that contained sunflower seeds, and 1 patient recalled feeding only dried corn. Only 1 patient recalled the brand of seed that they purchased. At the time of interview, no patients reported having any bird seed available for *Salmonella* testing. Seven (32%) patients, 6 of whom also owned birdfeeders, had contact with living or dead songbirds in the week before illness onset, including 1 patient who owned a pet sparrow and 1 who handled a wild finch. This number was higher than the percentage of healthy persons interviewed in the 2018–2019 FoodNet Population Survey who reported contact with pet or wild birds in the 7 days before the interview (4.6% of respondents; p = 0.001). 

Eighteen (82%) of the 22 patients owned pet dogs, and 7 (32%) owned pet cats. The percentage of patients reporting contact with dogs was significantly higher than for healthy persons interviewed in the FoodNet Population survey (68% of respondents; p = 0.03), but the percentage of patients reporting contact with cats was similar to that for healthy persons (39% of respondents; p = 0.86). Six patients reported their dog or cat was exposed to birds or birdfeeders. One patient reported their cat caught a wild bird and was subsequently hospitalized at a veterinary clinic with fever, diarrhea, and vomiting within 7 days before the patient’s illness onset; no isolates were obtained from either the cat or the bird. All 8 patients reporting no exposure to birdfeeders had a pet dog or cat. Of those patients, 2 reported their pets might have been exposed to wild birds in the week before illness onset, and 1 was the patient who handled the wild finch. Overall, 16 (73%) of 22 patients could be linked to wild bird exposure, either by direct contact with birds, contact with birdfeeders, or contact with companion animals that might have interacted with birds.

### Animal Testing

Environmental health partners and wildlife experts from involved states, academic research colleagues, and representatives from the United States Geological Service and SCWDS noted an increase in reports of sick and dead songbirds on private property and around birdfeeders. State public health officials and environmental health partners similarly indicated that a songbird fatality event, primarily involving finches, was occurring congruently with the outbreak timeline. In California, 2,048 individual reports of sick and dead birds were provided by the public and wildlife rehabilitation centers during November 1, 2020–May 6, 2021. Those reports included ≈2,440 sick and dead birds, and more than half were identified as pine siskins.

In total, Oregon, Washington, and California public health officials obtained 23 isolates of the outbreak strain from wild songbirds collected during December 2020–April 2021 (Figures 1, 2). The isolates were related within 0–12 cgMLST allele differences to each other and to the outbreak strain. In California, 66 songbirds from 11 counties were collected by the California Department of Fish and Wildlife’s Wildlife Health Laboratory. Salmonellosis was identified in 15 birds selected for postmortem examination at California Animal Health and Food Safety Laboratory System, including pine siskins (n = 9), Lawrence’s goldfinch (*S. lawrencei*; n = 1), lesser goldfinches (*S. psaltria*; n = 2), purple finches (*Haemorhous purpureus*; n = 2), and yellow-rumped warbler (*Setophaga coronate*; n = 1). SCWDS investigators additionally identified salmonellosis in 29 songbirds of 5 species: 15 pine siskins, 11 American goldfinches (*S. tristis*), 1 northern mockingbird (*Mimus polyglottos*), 1 northern cardinal (*Cardinalis cardinalis*), and 1 brown-headed cowbird (*Molothrus ater*). Most birds examined (27/29, 93%) died during January 22, 2021–March 23, 2021; the birds were identified in 8 states (Figure 1). Histopathological findings among birds examined at California Animal Health and Food Safety Laboratory System and SCWDS were similar and included ulcerative esophagitis and ingluvitis, consistent with songbird salmonellosis.

Oregon officials sequenced a *Salmonella* isolate from a wound sample from a pet dog’s mouth that was related within 0–12 cgMLST allele differences to the outbreak strain. The dog was treated at a veterinary hospital for surgical removal of stick fragments from its mouth. The dog’s owner reported that dead birds had been observed on their property and neighboring properties, but no human illnesses were linked to contact with the dog.

A CDC Investigation Notice was posted on April 1, 2021, that detailed the outbreak investigation and provided recommendations on cleaning and maintaining birdfeeders, preventing pets from being exposed to contaminated birdfeeders, how to report dead birds, and how to properly dispose of them (*31*). The notice was promoted on social media, news media, and through newsletters and partner outreach with wildlife experts and state partners.

## Discussion

We report a multistate outbreak of *Salmonella* infections in the United States linked to wild birds; a link was previously suspected to wild birds during another salmonellosis outbreak in 2009 (*6*). Internationally, human illness outbreaks have been associated with direct contact with wild birds (*8*,*11*,*23*). *Salmonella* has been isolated from healthy and ill wild birds across the United States for decades, and salmonellosis fatality events involving wild songbirds have occurred during that time (*6*,*10*,*14*). However, in the United States, the general prevalence of *Salmonella* in these wild bird populations has been difficult to estimate, presenting challenges in evaluating the magnitude of the risk for *Salmonella* spillover from wild birds to humans (*14*). The findings of this 2020–2021 outbreak exemplify the potential for spillover. Furthermore, genetically related clinical and wild bird isolates dating back to 2017 suggest sporadic human illnesses associated with wild bird contact might have occurred before the 2020–2021 outbreak (Figure 2), but exposure information is not available from historical patients to corroborate this.

Increased incidence of deaths of songbirds were reported during the course of the 2020–2021 human salmonellosis outbreak, although the outbreak strain was not confirmed in birds in all states with human cases. Environmental health experts speculated that the presence of birdfeeders likely attracted migratory songbirds, providing a source of exposure as the infection spread among individual songbirds. Pine siskins and other irruptive migrants vary their migration pattern partially in response to the seasonal availability of food and the availability of pine crops in their natural wintering grounds in Canada (*32*). Those migratory variances might contribute to increased congregation of birds around birdfeeders during some winters, which might result in the buildup of fecal material on and under feeders, especially if not routinely cleaned and disinfected. If birdfeeders become contaminated with *Salmonella*, they can serve as a source for human and wild bird infections (*11*,*33*). The presence of large numbers of pine siskins at feeders has been associated with avian salmonellosis outbreaks in the past, and as a highly gregarious species, pine siskins might play an important role in the transmission of *Salmonella* at feeders (*6*). Although recreational supplemental feeding practices might positively affect the health of some wild birds (*34*), the preponderance of evidence demonstrates that unsafe feeding practices can put birds at risk for infectious diseases, particularly during migration or disease epidemics (*16*). Factors that could increase infectious disease spread at feeders include feeder type (e.g., platform-style feeders that enable birds to perch on top of food or feeders and are constructed of materials that are difficult to clean); feeder location (e.g., placed in shady areas); providing various feed types that promote interspecies contact while feeding in close proximity; allowing feeders and surrounding areas to become soiled with feces, food waste, or animal carcasses; and providing food year-round regardless of external circumstances such as disease outbreaks or presence of sick and dead birds at the feeders (*33*).

Involvement of companion animals in this outbreak demonstrates the potential for illness transmission, either directly or indirectly, from wildlife or contaminated environments to pets and subsequently to humans. Multiple patients reported their pets had access to birdfeeders on their property or had direct contact with wild songbirds through predation. One patient’s cat reportedly became ill after catching a wild bird, and the outbreak strain was isolated from the mouth wound of a pet dog during the outbreak. Contact with wildlife is a known risk factor for *Salmonella* infection in companion animals (*25*), and a previous outbreak of salmonellosis in cats in Sweden was linked to wild bird predation (*24*). Ill cats in that outbreak also were thought to be the source of human illnesses (*24*). This situation has similarly occurred in the United States when pets infected with *Salmonella* treated at a single veterinary clinic were subsequently linked to salmonellosis among clinic staff members and owners of those pets (*35*); no single source of *Salmonella* was identified, but *Salmonella* Typhimurium was the causative serotype, similar to outbreaks previously linked to wild birds (*8*,*11*,*23*,*24*,*35*).

Overall, clinical salmonellosis is considered rare in companion animals (*25*), even though, at least among free-roaming domestic cats, predation of wildlife occurs often (*36*). Such pets either might be infrequently exposed to *Salmonella* through wildlife predation because of variability in infection prevalence among individual birds or might be *Salmonella* carriers rather than develop clinical signs of infection. Research into the prevalence of *Salmonella* carriage among pets that are in contact with wildlife would aid in measuring the salmonellosis risk that this behavior creates for pet owners.

The first limitation of this investigation is that not all patients were available or agreed to be interviewed, limiting the amount of data on potential *Salmonella* source exposures that could be explored and representativeness of the underlying population. Second, although testing of wild bird carcasses yielded the outbreak strain, no testing of patients’ homes, birdfeeders, or pets was performed to determine if the hypothesized links between humans and wildlife were definitively contaminated with the outbreak strain. Third, a limited number of wild birds reported during this outbreak were examined; thus, cause of death cannot be confirmed as salmonellosis in all cases. Isolating the outbreak strain from individual birds could represent merely the carriage of the organism and not necessarily the cause of disease and death. Fourth, this investigation was not able to determine what factors predominantly contributed to spread of *Salmonella* among wild birds. Additional information from patients (e.g., type of feeders used, feeder location, feeding practices) or testing of feeders or bird feed for *Salmonella* might have elucidated the sources of exposure for wild birds. Finally, identifying patients as part of an outbreak necessitates those ill persons to seek medical care, healthcare providers to order appropriate diagnostic testing, and positive test results to be reported to public health departments. Therefore, our investigation is likely an underestimate of the true number of persons that were affected by the outbreak strain (*1*) and an overestimate of the severity of illness.

This outbreak of *Salmonella* Typhimurium demonstrates the ongoing need to raise public awareness of the potential to acquire *Salmonella* from wild animals such as songbirds. To prevent birdfeeders and birdbaths from becoming a source of infection for birds and humans, use of birdfeeders and birdbaths should be reduced or eliminated, especially during an active disease outbreak. If birdfeeders and birdbaths are in use, a minimum of monthly cleaning and disinfection is recommended, but more frequent cleaning might be needed when feeders or baths become visibly soiled or when they are placed in shady areas where they remain moist (*31*,*37*). Persons should avoid direct contact with wild birds, particularly those that are visibly sick or dead, and should wash their hands after any contact with birds, feeders, or baths, even if wearing gloves (*31*). Similarly, pet owners should prevent their pets from contacting wild birds, birdfeeders, spilled seed, and birdbaths to reduce the potential for them to bridge the transmission of pathogens between wildlife and humans or to become infected with *Salmonella* themselves (*31*). Veterinarians and veterinary staff should be aware of the potential health risk for zoonotic pathogens like *Salmonella*, particularly when treating animals that frequently contact wildlife (*25*). Given the repeated detection of this outbreak strain over time and the periodic deaths associated with *Salmonella* in some wild birds, it is possible for further illness to arise in persons or their pets in the United States. As such, public health officials should continue to provide information about measures to prevent the transmission of illness between persons, pets, and wildlife and should continue to conduct similar investigations by using a One Health approach.
